# Inverted U-shaped correlation between serum low-density lipoprotein cholesterol levels and cognitive functions of patients with type 2 diabetes mellitus

**DOI:** 10.1186/s12944-021-01534-5

**Published:** 2021-09-12

**Authors:** Haoqiang Zhang, Wenwen Zhu, Tong Niu, Zheng Wang, Ke An, Wuyou Cao, Jijing Shi, Shaohua Wang

**Affiliations:** 1 Department of Endocrinology, Affiliated Zhongda Hospital of Southeast University, No.87 Dingjiaqiao Road, Nanjing, 210009 PR China; 2 School of Medicine, Southeast University, Nanjing, 210009 PR China

**Keywords:** Cholesterol, Low-density lipoprotein cholesterol, Lipids disorder, Type 2 diabetes mellitus, Cognitive function, Mild cognitive impairment

## Abstract

**Background:**

Low-density lipoprotein cholesterol (LDL-C) metabolic disorder is common in individuals with diabetes. The role of LDL-C in mild cognitive impairment (MCI) remains to be explored. We aim to investigate the associations between LDL-C at different levels and details of cognition decline in patients with type 2 diabetes mellitus (T2DM).

**Methods:**

Patients with T2DM (*n* = 497) were recruited. Clinical parameters and neuropsychological tests were compared between patients with MCI and controls. Goodness of fit was assessed to determine the linear or U-shaped relationship between LDL-C and cognitive function. The cut-off point of LDL-C was calculated. Correlation and regression were carried out to explore the relationship between cognitive dysfunction and LDL-C levels above and below the cut-off point.

**Results:**

Although no significant difference in LDL-C levels was detected in 235 patients with MCI, compared with 262 patients without MCI, inverted-U-shaped association was determined between LDL-C and Montreal Cognitive Assessment (MoCA). The cut-off point of LDL-C is 2.686 mmol/l. LDL-C (>2.686 mmol/l) is positively related to Trail Making Test B (TMTB) indicating executive function. LDL-C (<2.686 mmol/l) is positively associated with Clock Drawing Test (CDT) reflecting visual space function in patients with T2DM.

**Conclusion:**

Inverted U-shaped correlation was found between serum LDL-C and cognitive function in patients with T2DM. Despite that the mechanisms of different LDL-C levels involved in special cognitive dysfunctions remain incompletely clarified, excessive LDL-C damages executive function, while the deficient LDL-C impairs visual space function.

**Trial registration:**

ChiCTR-OCC-15006060.

**Supplementary Information:**

The online version contains supplementary material available at 10.1186/s12944-021-01534-5.

## Background

Cholesterol in brain accounts for more than 20% of total cholesterol in human body [1]. Cholesterol plays a necessary role in the formation and maintenance of synapses [2]. Increase in age could significantly reduce the synthesis of cholesterol in astrocytes, which may lead to the loss of synapses [3]. In patients with Alzheimer’s disease (AD), the levels of enzymes associated with cholesterol synthesis in the brain decreased significantly, and the process of cholesterol synthesis changed [4]. Interestingly, insulin-deficient diabetic mice exhibited reduction in brain cholesterol synthesis and cognitive decline. Additionally, neurons may be protected by increased levels of cholesterol in the medium from cholesterol deficiency [5]. However, studies reported an increase in cholesterol level (including its oxidative metabolites) in the cerebrospinal fluid (CSF) of patients with AD compared with the control [6–10]. Hypercholesterolemia is a common risk factor of AD [11, 12] and MCI [13], a stage between normal cognitive function and AD dementia [14].

Brain cholesterol is mainly synthesized by astrocytes [15–17], but cells in the brain can also obtain cholesterol binding apolipoproteins from adjacent tissues via a receptor-mediated form [18]. Most cholesterol in the whole body is synthesized in the liver and supplied to other organs. The liver also metabolizes excess cholesterol in preparation for secretion into intestines and excretion in feces [19, 20]. In our previous study, we found elevated levels of total cholesterol (TC) and LDL-C in plasma, and observed cognitive dysfunction in LDLR knockout mice [21]. Thus, serum cholesterol homeostasis may reflect the cholesterol balance of specific organs to a certain degree. We hypothesized that the heavy cholesterol loads in blood circulation or the brain may lead to cognitive impairment. Indeed, ATP-binding cassette A1 transporter R219K variants altered the outflow of cholesterol from the brain and the modified serum lipid profiles may contribute to cognition dysfunction in patients with T2DM [22]. However, reduced cholesterol synthesis contributes to neuronal damages and lead to cognitive dysfunction in insulin-deficient diabetic mice [23].

Although the relationship between LDL-C and cognitive function have been well discussed, the disorder of lipids does not appear alone. The prevalence of T2DM increased to 9.3 and 11.2% in global [24] and Chinese populations [25] respectively. Additionally, T2DM significantly increased the risk of cognitive impairment [26]. For the large population of T2DM patients with obesity or hypercholesterolemia, research on the relationship between cholesterol and cognitive function seems very important. Scholars have focused on diabetic cognition decline [27, 28]. However, the roles of different cholesterol levels in patients with T2DM and MCI should be further explored. The present work was designed to confirm the correlations between cholesterol profiles and cognition function in diabetic patients.

## Methods

### Ethics

Patients at Department of Endocrinology, Affiliated Zhongda Hospital of Southeast University were enrolled in this study. Every volunteer provided an informed consent before joining the research. The present work was approved by the Research Ethics Committee of our institution (approval no. of ethics committee: 2013ZDSYLL040.0).

### Subjects and groups

A total of 497 patients aged 40–80 years (235 with MCI and 262 without MCI) were recruited between August 2013 and December 2019. The inclusion criteria were as follows: 1) all patients were Han Chinese (right-handed) and had education level of at least 6 years; and 2) the duration of T2DM was more than 3 years. The exclusion criteria were as follows: 1) acute diabetic complications; 2) acute cardiovascular or cerebrovascular diseases; 3) history of brain diseases; 4) abuse of alcohol or drug (in near 2 months); 5) thyroid dysfunction; 6) other illnesses, such as serious infection or surgery, cancer, or anemia (in near 6 months), that influence (or potentially influence) cognitive function, neuropsychological test, or measurement of blood samples; 7) visual or hearing loss; and 8) severe cognitive decline (out of the range of MCI). All individuals with diabetes had disease duration of more than 3 years. MCI was diagnosed by the criteria from the MCI Working Group of the European Consortium for Alzheimer’s Disease [29].

### Clinical data

Data on age, sex, and education level as well as durations of T2DM and high blood pressure (HBP) were collected. Smoking history data were also gathered. When the patients obtained medical care in Zhongda Hospital, the levels of glycosylated hemoglobin (HbA1c), triglyceride (TG), total cholesterol (TC), LDL-C and high-density lipoprotein (HDL-C) were measured in blood samples by the Laboratory Center of our institution. Body mass indices (BMI) were calculated by height and body weight. The Laboratory Center of our institution implemented quality control standards according to the Chinese Laboratory Quality Control.

### Neuropsychological tests

MoCA scores were used to assess global cognitive abilities. One score was added if the years of education were less than 12. Digit Span Test (DST), Verbal Fluency Test (VFT) and TMTB were conducted to evaluate patients’ executive functions. CDT was used to analyze for visual space function. Trail Making Test-A (TMTA) test was performed to assess information processing speed function. The enrolled patients with T2DM were divided into two groups: MCI group (< 26) and Non-MCI group (≥ 26). MoCA score was calculated according to a previously reported method [30]. Additional information can be found at the website (https://www.mocatest.org/). CDT was included in the performance of MoCA. DST [31] and VFT [32] as well as TMTA and TMTB [33] were performed according to previous studies.

### Statistical analysis

Statistical analyses were conducted by SPSS 20.0. Student’s t test, nonparametric Mann-Whitney U and Chi-squared test were performed for normally distributed, asymmetrically distributed, and binary variables, respectively. Curve fitting and line fitting were conducted to detect the relationship between LDL-C (or TC) and MoCA by curve estimation. Cut-off point (= − b/2a) was calculated for U-shaped curve (with a regression equation: Y = aX^2^ + bX + c). Pearson or partial correlation analysis was conducted to observe the association between LDL-C (or TC) and MoCA without or with adjustment by age, gender, education and HBP duration respectively. Binary logistic regression was used to explore the independent risk factors of MCI. Multiple linear regression was employed to investigate variables influencing TMTB and CDT in different populations. *P* < 0.05 was defined as statistical significance.

## Results

### Baseline data of patients with T2DM in MCI or non-MCI group

Although increased cholesterol is a common risk factor of AD and our previous study showed that poorly controlled LDL-C is related to executive function in diabetic patients [13], no significant difference in TC and LDL-C levels were found among T2DM patients with or without MCI (*P* = 0.196 and 0.475 respectively). Age, gender and education levels varied among the patients in the two groups (All *P*<0.001). Interestingly, the HBP duration of patients with MCI was longer than that of patients without MCI (*P* = 0.006). The HbA1c was higher in patients with MCI than in patients without MCI, but the difference was not significant (*P* = 0.090). MoCA, DST, VFT, CDT, TMTA, and TMTB were significantly different between the two groups of patients (All *P*<0.001) (Table 1).

**Table 1:** Demographic, clinical and cognitive characteristics of T2DM patients with or without MCI

	All individuals (*n* = 497)	MCI (*n* = 235)	Non-MCI (*n* = 262)	*P* value
Age (year)	60 (53–66)	62 (56–68)	58 (52–64)	<0.001^b^*
Female (n, %)	207 (41.6)	117 (49.8)	90 (34.4)	<0.001^c^*
Education	10 (9–12)	9 (6–12)	12 (9–12)	<0.001^b^*
BMI (Kg/m^2^)	24.77 (22.77–27.04)	24.57 (22.77–27.12)	24.80 (22.75–26.86)	0.869^b^
DM Duration (year)	10 (5–14)	10 (5–15)	10 (5–13)	0.352^b^
HBP Duration (year)	3 (0–10)	5 (0–15)	1 (0–10)	0.006^b^*
Smoking (n, %)	178 (35.8)	79 (33.6)	99 (37.8)	0.333^c^
Statins (n, %)	265 (53.3)	133 (56.6)	132 (50.4)	0.166^c^
Other Antilipidemic Drugs (n, %)	24 (4.8)	15 (6.4)	9 (3.4)	0.126^c^
HbA1c (%)	8.60 (7.20–10.30)	8.80 (7.50–10.50)	8.40 (7.10–10.10)	0.090^b^
TG (mmol/l)	1.48 (1.04–2.27)	1.53 (1.00–2.38)	1.48 (1.05–2.18)	0.599^b^
TC (mmol/l)	4.60 ± 1.15	4.67 ± 1.24	4.54 ± 1.07	0.196^a^
HDL-C (mmol/l)	1.15 (1.00–1.34)	1.16 (1.01–1.34)	1.15 (0.98–1.32)	0.300^b^
LDL-C (mmol/l)	2.82 (2.29–3.43)	2.85 (2.23–3.63)	2.81 (2.32–3.24)	0.475^b^
LDL-C/HDL-C	2.43 (2.00–2.95)	2.43 (1.98–3.03)	2.42 (2.01–2.91)	0.819^b^
MoCA	26 (23–27)	22 (20–24)	27 (26–28)	<0.001^b^*
DST	12 (10–13)	11 (9–12)	12 (11–14)	<0.001^b^*
VFT	16 (14–19)	15 (13–18)	18 (15–21)	<0.001^b^*
CDT	4 (3–4)	3 (2–4)	4 (3–4)	<0.001^b^*
TMTA	60 (49–83)	71 (57–95)	55 (45–67)	<0.001^b^*
TMTB	160 (123–205)	185 (145–236)	135 (106–178)	<0.001^b^*

*Abbreviations*: *BMI* Body mass index, *DM* Diabetes mellitus, *HBP* High blood pressure, *FPG* Fasting plasma glucose, *TG* Triglycerides, *TC* Total cholesterol, *LDL-C* Low density lipoprotein cholesterol, *HDL-C* High density lipoprotein cholesterol, *MoCA* Montreal Cognitive Assessment, *DST* Digit Span Test, *VFT* Verbal Fluency Test, *CDT* Clock Drawing Test, *TMTA* Trail Making Test-A, *TMTB* Trail Making Test-B, *MCI* Mild cognitive impairment

The data are presented as n (%), or the median (inter-quartile range) unless otherwise specified

^a^Student’s t test was employed for normally distributed variables

^b^The Mann-Whitney U test was employed for asymmetrically distributed variables

^c^The Chi-square test was employed for categorical variables

*P < 0.05

### Association between MoCA and TC (or LDL)

**Fig. 1:**
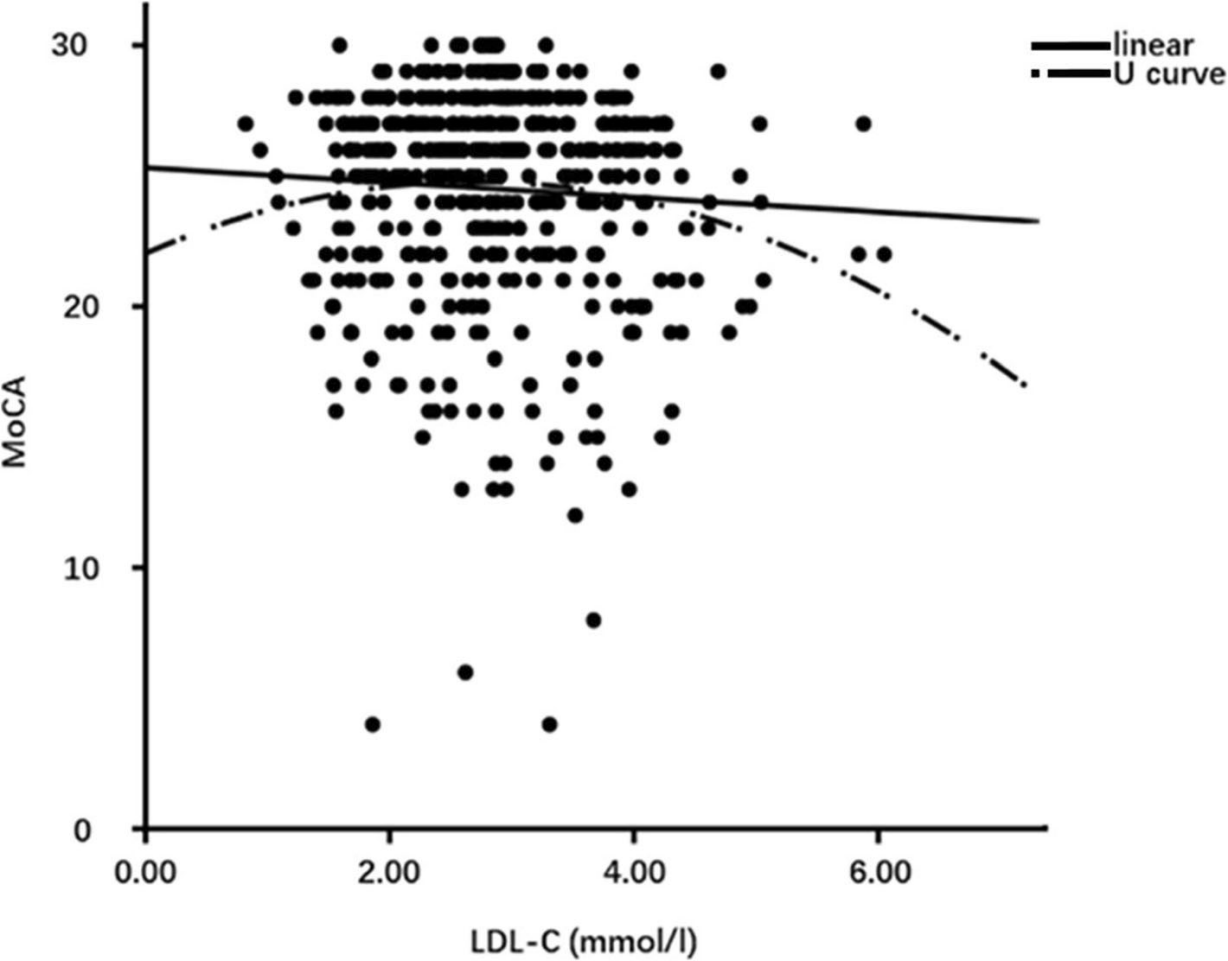
Comparison of linear and U curve association between LDL-C and MoCA. Cut-off point of LDL-C: 2.686 mmol/l. Abbreviations: LDL-C, low density lipoprotein cholesterol; MoCA, Montreal Cognitive Assessment

Despite the lack of statistical significance in the difference of TC and LDL-C levels between patients with or without MCI, the relationship between MoCA and TC (or LDL-C) was analyzed. No correlation was found between TC (or LDL-C) and MoCA by Pearson association (R = -0.056, *P* = 0.216 or R = -0.058, *P* = 0.197). Although the association between TC and MoCA was not detected, the association between LDL-C levels and MoCA scores were observed by partial correlation adjusted by age, gender, education and HBP duration (R = -0.087, *P* = 0.054 and R = -0.099, *P* = 0.027) (Supplementary Table 1). Further binary logistic regression analysis indicated that LDL-C was not a risk factor of MCI in T2DM patients (*P* = 0.062, OR = 0.803) (Supplementary Table 2). Linear and U-shaped curve were compared to further explore the relationship between cholesterol and cognition decline. Interestingly, we detected an inverted-U-shaped cueve rather than a linear association between LDL-C and MoCA (*P* = 0.044 vs *P* = 0.196) (Fig. 1 and Supplementary Table 4). However, no significant linear association or U-shaped association was detected between TC and MoCA (all *P*>0.05) (Supplementary Figure 1 and Supplementary Table 3). Additionally, the cut-off point of LDL-C was calculated to be 2.686 mmol/L.

### Association between cognitive function and LDL-C in different levels

The associations between LDL-C and neuropsychological tests results above or below the cut-off point were analyzed by partial correlation adjusted by age, gender, education, HBP duration and HbA1c level to further explore the relationship between LDL-C and cognitive function. Although MoCA was not associated with LDL-C below 2.686 mmol/L, it was related to LDL-C above 2.686 mmol/L (R = -0.143, *P* = 0.015). Moreover, LDL-C (<2.686 mmol/L) was associated with CDT (R = 0.151, *P* = 0.033), while LDL-C (>2.686 mmol/L) was related to TMTB (R = 0.180, *P* = 0.002) (Table 2).

**Table 2:** Association between cognitive function and LDL-C in different levels

	LDL-C ≤ 2.686 mmol/l		LDL-C>2.686 mmol/l	
	R	*P*	R	*P*
MoCA	0.027	0.704	-0.143	0.015*
DST	0.111	0.119	−0.105	0.076
VFT	− 0.031	0.661	− 0.056	0.340
CDT	0.151	0.033*	−0.065	0.270
TMTA	0.044	0.536	−0.001	0.989
TMTB	0.002	0.981	0.180	0.002*

*Abbreviations*: *LDL-C* Low density lipoprotein cholesterol, *MoCA* Montreal Cognitive Assessment, *DST* Digit Span Test, *VFT* Verbal Fluency Test, *CDT* Clock Drawing Test, *TMTA* Trail Making Test-A, *TMTB* Trail Making Test-B

*P < 0.05

### Binary logistic regression for risk factors of MCI

Higher LDL-C level (above the cut-off point), not lower LDL-C level (below the cut-off point), was an independent risk factor of MCI in diabetic patients (*P* = 0.012, OR = 0.581) (Table 3).

**Table 3:** Binary logistic regression analysis for MCI risk in patients with different LDL-C levels

		LDL ≤ 2.686 mmol/l				LDL>2.686 mmol/l		
	*P*	OR	95%CL of OR		*P*	OR	95%CL of OR	
			Lower	Higher			Lower	Higher
Age	0.275	0.978	0.941	1.017	0.032*	0.966	0.936	0.997
Gender	0.865	0.947	0.503	1.781	0.143	0.677	0.401	1.140
Education	<0.001*	1.232	1.113	1.363	<0.001*	1.214	1.119	1.316
HBP Duration	0.173	0.978	0.947	1.010	0.152	0.980	0.953	1.008
LDL-C	0.225	1.602	0.748	3.430	0.012*	0.581	0.381	0.886

*Abbreviations*: *LDL-C* Low density lipoprotein cholesterol, *MCI* Mild cognitive impairment, *HBP* High blood pressure

*P < 0.05

### Multiple linear regression for the influence of LDL-C

Multiple linear regression was conducted to investigate the relationship between CDT and LDL-C below 2.686 mmol/L as well as TMTB and LDL-C above 2.686 mmol/L given that the partial correlation analysis showed the association between LDL-C and neuropsychological tests above or below 2.686 mmol/L. Interestingly, LDL-C is related to TMTB (*P* = 0.002, β = 19.393) (LDL-C>2.686 mmol/L), and associate with CDT (*P* = 0.033, β = − 0.320) (LDL-C<2.686 mmol/L) as age, gender, education, HBP duration, HbA1c and LDL-C were entered as independent variables (Tables 4 and 5).

**Table 4:** Multiple linear regression analysis of the influence of LDL-C (>2.686 mmol/l) on TMTB

	*P*	β	95% CL of β	
			Lower	Higher
Age	<0.001*	2.878	1.980	3.777
Gender	0.304	8.109	−7.399	23.618
Education	<0.001*	−5.877	−7.977	−3.777
HBP duration	0.108	0.683	−0.151	1.516
LDL-C	0.002*	19.393	7.051	31.735

*Abbreviations*: *LDL-C* Low density lipoprotein cholesterol, *TMTB* Trail Making Test-B, *AVLT-DR* Auditory Verbal Learning test-delayed recall, *HBP* High blood pressure

*P < 0.05

**Table 5:** Multiple linear regression analysis of the influence of LDL-C (<2.686 mmol/l) on CDT

	*P*	β	95% CL of β	
			Lower	Higher
Age	0.219	0.009	−0.006	0.025
Gender	0.077	−0.224	−0.472	0.025
Education	0.004*	0.051	0.016	0.086
HBP duration	0.255	−0.007	−0.019	0.005
LDL-C	0.033*	0.320	0.026	0.614

Age, gender, education, HBP duration and LDL-C were entered as independent variables

*Abbreviations*: *LDL-C* Low density lipoprotein cholesterol, *CDT* Clock Drawing Test, *HBP* High blood pressure

*P < 0.05

## Discussion

Hyperglycemia is a well-recognized risk factor of cognition decline. Uncontrolled HbA1c levels were detected in our previous studies [34–36] and other works [37]. Although HbA1c levels were not significantly different, higher HbA1c levels were measured in patients with MCI than in patients without MCI in the present study.

Obesity commonly exists in patients with T2DM, who may easily suffer from hypercholesterolemia. Increasing number of studies have shown that hypercholesterolemia is a risk factor of cognition decline [38–40]. However, the results are conflicting. Although increased TC or LDL-C levels were observed in diabetic patients with MCI, compared with those without MCI, no statistical significance was found. Despite that the association between LDL-C and MoCA was detected by partial correlation adjusted by age, gender, education and HBP duration, LDL-C was not an independent risk factor of MCI, which is assessed by binary logistic regression analysis in all patients.

These results confuse us. The relationship between cholesterol and cognitive function may not be a linear association because cholesterol is an essential element of the membrane of neurons and play a necessary role in the function of synapses. Extremely low cholesterol levels may damage cognitive function because increase in cholesterol could promote the survival of neurons. Moreover, high cholesterol levels (in the high normal range) may have a protective effect on cognitive function in old Chinese people [41]. The U-shaped relationship may exist between cholesterol levels and cognitive function. In other words, cholesterol deficiency destroys the structural integrity of neurons and affects their normal function. However, as a metabolic disorder, hypercholesterolemia also contributes to cognitive impairment by directly damaging the nervous system (for the modification of cholesterol including but not limited to oxidized cholesterol) or declining vascular cognition [42, 43].

To confirm the above hypothesis, we compared the linear and U-shaped relationship curves between cholesterol and cognitive function by goodness of fit. Although the U-shaped association between MoCA and TC was not confirmed, the U-shaped relationship between LDL-C and MoCA was found. Additionally, the cut-off point of LDL-C (2.686 mmol/L) was calculated. Partial correlation was conducted with adjustment for age, gender, education and HBP duration to assess the relationship between cognitive function (including details of cognitive function and global cognitive function) and LDL-C above/below the cut-off point. At LDL-C above 2.686 mmol/L, MoCA was associated with serum LDL-C. Moreover, increased LDL-C was found to be an independent risk factor of MCI in T2DM patients at this range by binary logistic regression. This finding is consistent with our previous work [13]. Xia et al suggested that uncontrolled cholesterol levels are associated with MCI in diabetic patients in a study with fMRI. Additionally, the brain damage of elevated cholesterol was found in our animal study [21]. These results partly confirm that hypercholesterolemia, especially elevated LDL-C is a risk factor of cognition decline and explain the conflict of previous studies in clarifying the relationship between cholesterol levels and cognitive function [41, 44–47].

Despite changes in Aβ metabolism and transformation in the brain, hypercholesterolemia tends to increase the risk of vascular dementia [48], while cholesterol deficiency may affect cognitive impairment by injuring the synaptic function [2]. Additional correlation analyses between neurological tests results and cholesterol levels were performed to further study the details of the damage of hypercholesterolemia to cognitive dysfunction. LDL-C level was positively associated with execution function in patients with hypercholesteremia. Similarly, our latest results show that hypercholesteremia, especially increased LDL-C may influence the executive function of patients with T2DM [49]. Additionally, Xia et al demonstrated the disordered functional connectivity of bilateral hippocampus-middle frontal gyrus in diabetic patients with poorly controlled LDL-C, and associated with executive function [13]. Although MoCA was not associated with LDL-C below 2.686 mmol/L in the present work, the association between visual space function and decrement of LDL-C was detected by partial correlation. Moreover, LDL-C (<2.686 mmol/L) was positively associated with the visual space function, independent from age, gender, education, HBP duration and HbA1c in the multiple linear regression analysis. These results show that cholesterol should not be decreased to exceptionally low levels, especially for patients with risks of cognitive impairment.

### Comparisons with other studies and what does the current work add to the existing knowledge

In this research, we investigated the relationship between cognitive function and LDL-C levels in different populations (with LDL-C above or below the cut-off point) with T2DM. Most previous studies indicated that elevated LDL-C levels is a risk or protective factors in specific individuals. Here, we found different levels of cognitive dysfunction in T2DM patients with different LDL-C levels. This novel findings provids an evidence for the management of LDL-C of T2DM patients, especially those with MCI.

### Study strengths and limitations

In this work, we first demonstrated the inverted-U-shaped correlation between serum LDL-C level and cognitive functions among T2DM patients. Additionally, elevated LDL-C above the cut-off point is a risk factor of MCI. These results show that an appropriate level of LDL-C is beneficial for the cognitive function of T2DM patients. Since LDL-C was involved in this work, statins and other lipid-lowering drugs were recorded. However, names, dozes, and usage duration were not considered in subgroup analysis to avoid the decrease in the effectiveness of statistical results from limited numbers of participants. In this metabolite study, we only focused on the levels of cholesterol and medicine involved in cholesterol metabolism, but failed to investigate relationship between diet and cholesterol, which should be considered in the further research. Despite that elevated LDL-C is a risk factor of MCI in T2DM patients, and associated with executive function, the mechanism remains unclear and need further works, especially for the mechanisms involved in oxidized cholesterol. Additionally, unmatched patients were recruited in this cross-sectional study. Although these unmatched variables were adjusted, they may result in the small correlation coefficients. Finally, the present study only explained associations between different levels of LDL-C and cognitive function performance, but their causal relationship needs to be further clarified by cohort studies and basic experiments.

## Conclusion

To our best knowledge, this study is the first to report an inverted-U-shaped correlation between serum LDL-C and global cognitive function in T2DM patient. Appropriate cholesterol level, especially for LDL-C, is beneficial to maintaining normal cognitive function. Extremely high or low LDL-C levels are not desirable for cognitive function in T2DM patients. Additionally, excessive cholesterol levels damage the executive function, while deficient LDL-C impairs visual space function. Extremely high levels of LDL-C should be well controlled for the benefit of cognitive function in T2DM patients. Additionally, LDL-C should not be decreased to very low levels, especially for T2DM patients with cognition decline.

## Supplementary Information

**Additional file 1: Supplementary Table 1.** Association between MoCA and TC (or LDL-C). **Supplementary Table 2.** Binary logistic regression analysis for MCI risk in all patients. **Supplementary Table 3.** Comparison of line fitting and U-shaped curve fitting assessing the association between TC and MoCA. **Supplementary Table 4.** Comparison of line fitting and U-shaped curve fitting assessing the association between LDL-C and MoCA. **Supplementary Figure 1.** Comparison of linear and U curve association between TC and MoCA. Abbreviations: TC, Total cholesterol; MoCA, Montreal Cognitive Assessment.

## Data Availability

The datasets analyzed are available from the corresponding author on reasonable request.
