# Supplementary material for: Inverted U-shaped correlation between serum low-density lipoprotein cholesterol levels and cognitive functions of patients with type 2 diabetes mellitus
Source: Lipids Health Dis. 2021 Sep 12;20:103. doi: 10.1186/s12944-021-01534-5 (PMC8436464; doi:10.1186/s12944-021-01534-5)
Supplement: Supplementary file 1 — Additional file 1: Supplementary Table 1. Association between MoCA and TC (or LDL-C). Supplementary Table 2. Binary logistic regression analysis for MCI risk in all patients. Supplementary Table 3. Comparison of line fitting and U-shaped curve fitting assessing the association between TC and MoCA. Supplementary Table 4. Comparison of line fitting and U-shaped curve fitting assessing the association between LDL-C and MoCA. Supplementary Figure 1. Comparison of linear and U curve association between TC and MoCA. Abbreviations: TC, Total cholesterol; MoCA, Montreal Cognitive Assessment. [file 12944_2021_1534_MOESM1_ESM.docx]

Supplementary table1: Association between MoCA and TC (or LDL-C)

|  | TC | | LDL-C | |
| --- | --- | --- | --- | --- |
|  | R | *P* | R | *P* |
| Model 1 | -0.056 | 0.216 | -0.058 | 0.197 |
| Model 2 | -0.087 | 0.054 | -0.099 | 0.027* |

1 Pearson association between MoCA and TC (or LDL-C)

2 Partial correlation between MoCA and TC (or LDL-C) between adjusted by age, gender, education and HBP duration

**P*<0.05

Abbreviations: MoCA, Montreal Cognitive Assessment; TC, Total cholesterol; LDL, low density lipoprotein cholesterol; HBP, high blood pressure.

Supplementary table2: Binary logistic regression analysis for MCI risk in all patients

|  | *P* | OR | 95%CL of OR | |
| --- | --- | --- | --- | --- |
|  |  |  | Lower | Higher |
| Age | 0.010* | 0.969 | 0.946 | 0.993 |
| Gender | 0.181 | 0.762 | 0.512 | 1.135 |
| Education | ＜0.001* | 1.222 | 1.147 | 1.301 |
| HBP Duration | 0.053 | 0.980 | 0.959 | 1.000 |
| LDL-C | 0.062 | 0.803 | 0.637 | 1.011 |

**P*<0.05

Abbreviations: LDL-C, low density lipoprotein cholesterol; MCI, mild cognitive impairment; HBP, high blood pressure.

Supplementary table3: Comparison of line fitting and U-shaped curve fitting assessing the association between TC and MoCA

|  | R2 | *P* | A | B1 | B2 |
| --- | --- | --- | --- | --- | --- |
| Line | 0.003 | 0.216 | 25.409 | -0.195 |  |
| U-shape | 0.004 | 0.406 | 24.408 | 0.243 | -0.045 |

Abbreviations: TC, total cholesterol; MoCA, Montreal Cognitive Assessment;

Supplementary table4: Comparison of line fitting and U-shaped curve fitting assessing the association between LDL-C and MoCA

|  | R2 | *P* | A | B1 | B2 |
| --- | --- | --- | --- | --- | --- |
| Line | 0.003 | 0.197 | 25.323 | -0.283 |  |
| U-shape | 0.013 | 0.044* | 22.035 | 2.052 | -0.382 |

**P*<0.05

Abbreviations: LDL-C, low density lipoprotein cholesterol; MoCA, Montreal Cognitive Assessment;


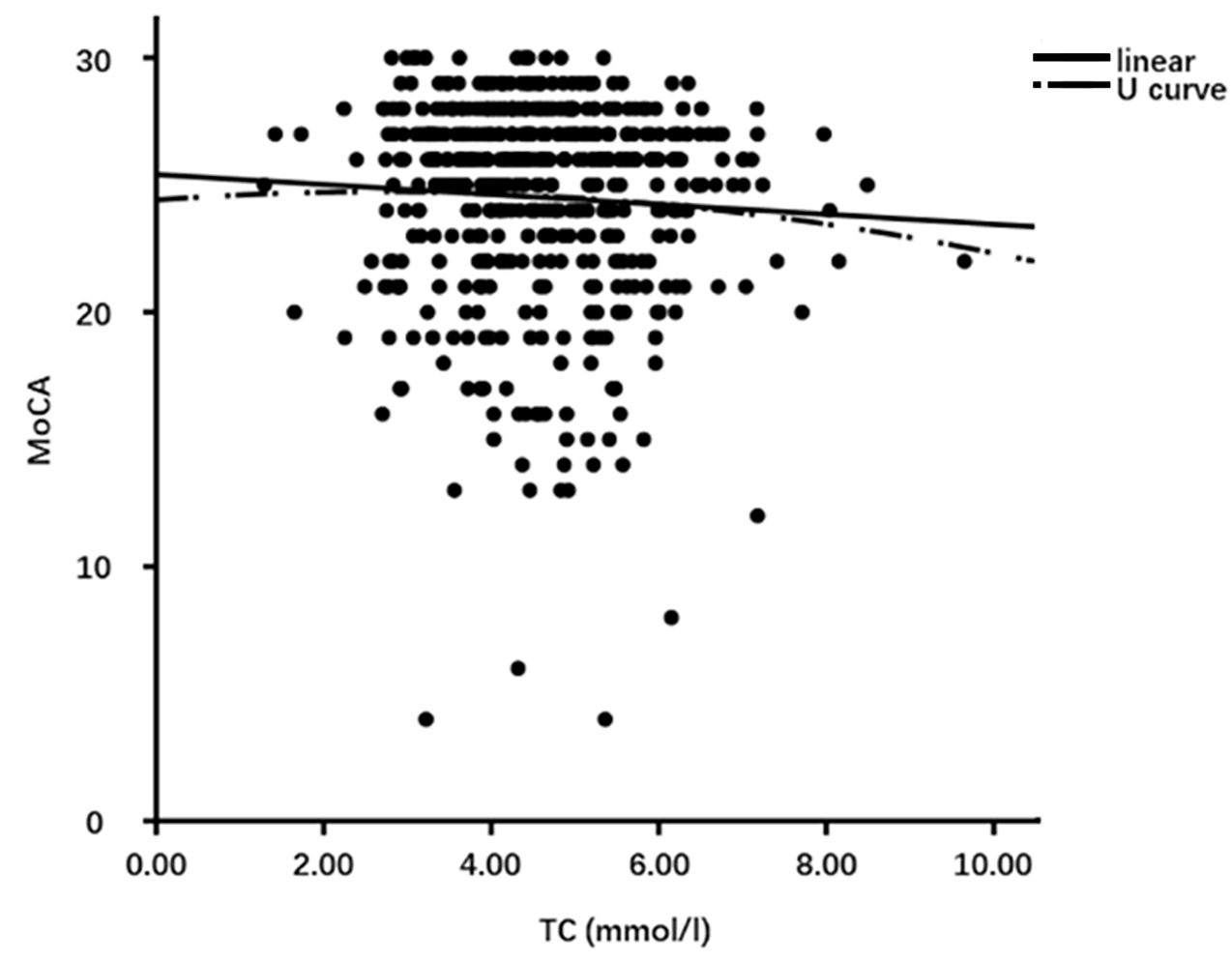


Supplementary Figure1: Comparison of linear and U curve association between TC and MoCA. Abbreviations: TC, Total cholesterol; MoCA, Montreal Cognitive Assessment
